# RURAL LIBRARIES AS CONTESTED SPACES OF OLDER VOLUNTARISM IN AGING RURAL COMMUNITIES

**DOI:** 10.1093/geroni/igz038.064

**Published:** 2019-11-08

**Authors:** Amber Colibaba, Mark Skinner

**Affiliations:** Trent University, Peterborough, Ontario, Canada

## Abstract

Recent efforts to better understand voluntarism as fundamental to how rural communities are meeting the challenges of population ageing have highlighted ageing rural volunteers, and the attendant burden of older voluntarism, as key issues for ageing in place of rural residents and ageing rural community sustainability. Drawing on a case study of a volunteer-based rural library in Ontario, Canada, this study examines the experiences of older volunteers, the challenges of sustaining volunteer programs, and the implications of older voluntarism for rural community development. Findings from interviews and focus groups with library volunteers, staff, board members and community stakeholders demonstrate how the experiences of older volunteers and challenges of older voluntarism affect rural community development. The results reveal how participation, well-being, conflict and territoriality associated with older voluntarism contributes to ‘contested spaces of older voluntarism’ whereby older volunteers negotiate their rights and responsibilities associated with ageing and volunteering in rural communities.

